# Validation of the Rational-Experiential Inventory (REI-40) in Brazilian Portuguese

**DOI:** 10.3390/bs16060885

**Published:** 2026-06-01

**Authors:** Julio Cesar de Aguiar, Benjamin Miranda Tabak

**Affiliations:** School of Public Policy, Government and Business, Getulio Vargas Foundation, FGV EPPG, Brasília 70830-051, DF, Brazil; julio.aguiar@fgv.br

**Keywords:** Rational-Experiential Inventory, dual-process theory, thinking styles, psychometric validation, cognitive styles

## Abstract

(1) Background: The Rational-Experiential Inventory (REI-40) is a widely used instrument for assessing individual differences in rational and experiential thinking styles based on Cognitive-Experiential Self-Theory (CEST). Despite its international prominence, no comprehensive validation existed for Brazilian Portuguese populations. (2) Methods: This study validated the REI-40 for Brazilian Portuguese through confirmatory factor analysis (CFA) estimated with a robust weighted least squares estimator (WLSMV) appropriate for ordinal items, comparison of competing structural models, internal-consistency and validity testing, and tests of measurement invariance across sex, education, and age, in a sample of 464 legal professionals from Brazil’s Advocacy-General of the Union. (3) Results: The hypothesized four-factor, 40-item model showed acceptable fit (χ^2^(734) = 1815.8, CFI = 0.902, TLI = 0.896, RMSEA = 0.056, SRMR = 0.080) and clearly outperformed unidimensional and two-factor alternatives. McDonald’s omega indicated good internal consistency for all subscales (ω = 0.79–0.87). Measurement invariance across sex, education, and age was supported at the configural and metric levels. Validity evidence included positive correlations between rational subscales and CRT-7 (r = 0.24–0.31) and weak or negative correlations for experiential subscales. (4) Conclusions: The Brazilian Portuguese REI-40 demonstrates adequate psychometric properties for measuring thinking styles in professional populations.

## 1. Introduction

Individual differences in cognitive processing styles have profound implications for decision-making, problem-solving, and professional performance across diverse contexts. Understanding how individuals balance analytical reasoning with intuitive judgment has become increasingly important in organizational, educational, and clinical settings. The Rational-Experiential Inventory (REI-40; Pacini & Epstein, 1999) represents one of the most widely used instruments for assessing these differences, grounded in Seymour Epstein’s Cognitive-Experiential Self-Theory (CEST). However, despite its international prominence and extensive use in cross-cultural research, no comprehensive validation of the REI-40 existed for Brazilian Portuguese populations before this research, limiting its application within South America’s largest psychological research community.

The present study addresses this gap by providing the first systematic psychometric evaluation of the REI-40 in Brazilian Portuguese. This validation is particularly important given Brazil’s unique cultural context, which combines Latin American collectivistic values with increasing emphasis on analytical thinking in professional domains. Furthermore, validation with a sample of legal professionals provides important evidence of the instrument’s utility in high-stakes professional contexts, where both analytical and intuitive processing contribute to effective performance.

This research is an effort to bridge a clear gap in psychometrics and cross-cultural literature through offering the first-ever systematic validation of the REI-40 in Brazil. Based on the principles of CEST, the paper contributes to the development of dual-process theory by empirically verifying the theoretical autonomy of the rational subsystems, namely, deliberative and analytical, and the experiential subsystems, including intuitive and holistic, in an environment that features high professional efficacy. The results provide evidence for the assumption that intuition does not equal the absence of analysis but represents a separate process that is independent of intelligence, validating the four-factor model and establishing convergent and discriminant validity by correlating with the CRT-7. Hence, in addition to contributing to the literature through translation and adaptation, the paper offers a reliable tool for analyzing the interaction among those subsystems in decision-making and for designing appropriate interventions within organizational and public policy frameworks in South America.

### 1.1. Theoretical Foundation: Cognitive-Experiential Self-Theory

Cognitive-Experiential Self-Theory posits that human information processing operates through two distinct but interacting systems (Epstein, 2003, 2014). The rational system engages in conscious, analytical processing, characterized by deliberate thought processes, verbal reasoning, and the explicit use of knowledge. This system is slower, more effortful, and has limited processing capacity, but excels in logical analysis and systematic problem-solving (Epstein et al., 1996; Epstein, 2014). The rational system operates according to logical rules, is relatively affect-free, and requires justification through logic and evidence.

In contrast, the experiential system operates primarily at preconscious levels through automatic, intuitive processing. It relies on emotions, imagery, and past experiences to guide judgments and actions, functioning faster and with greater processing capacity than the rational system (Shiloh et al., 2002; Salas-Auvert & Felgoise, 2003). This system connects to implicit learning, gut feelings, and heuristic decision-making processes, enabling rapid responses to complex situations (Phillips et al., 2016). The experiential system is holistic, associates events through similarity and contiguity, and is experienced as self-evidently valid.

The dynamic interaction between these systems, sometimes synergistic and sometimes conflicting, produces individual differences in cognitive style that influence behavior across numerous domains (Epstein, 2003, 2008). Importantly, CEST proposes that both systems operate in parallel and that effective functioning often requires integration of both processing modes. Understanding this dual-process framework has significant implications for therapeutic intervention, educational design, health communication, and organizational effectiveness (Fletcher et al., 2011; Dunlop et al., 2011; Cerni et al., 2014).

### 1.2. The REI-40: Structure and Psychometric Properties

The REI-40 operationalizes CEST through four distinct subscales that capture both ability and engagement dimensions of rational and experiential processing (Pacini & Epstein, 1999). Rational Ability (RA) measures confidence in logical and analytical thinking capabilities, assessing individuals’ perceptions of their analytical problem-solving skills. Rational Engagement (RE) assesses preference for and enjoyment of analytical thinking, capturing the motivational component of rational processing. Similarly, Experiential Ability (EA) assesses confidence in intuitive and experiential thinking, while Experiential Engagement (EE) measures preference for and reliance on feelings and intuition in decision-making.

Research has demonstrated the REI-40’s utility across diverse applications. In clinical contexts, physicians with higher experiential scores exhibited different decision-making patterns compared to those who favored rational processing (Calder et al., 2012). Educational research has revealed how thinking style preferences influence learning approaches and academic performance (Norris & Epstein, 2011). In organizational settings, the instrument has been linked to leadership effectiveness, team dynamics, and managerial decision-making (Cerni et al., 2014; Curtis & Wee, 2021). The REI-40 has also been used in research on health professionals’ decision-making (McLaughlin et al., 2014) and clinical judgment (Sladek et al., 2010).

Cross-cultural applications of the REI-40 have revealed important considerations for validation studies. Research has found gender and age differences in REI-40 scores across different populations (Calder et al., 2012; Sladek et al., 2010), while some studies have challenged the assumption of complete independence between rational and experiential dimensions (Hodgkinson et al., 2009). Factor analytic studies have generally supported the four-factor structure, though fit indices vary across samples (Witteman et al., 2009). These findings underscore the importance of population-specific validation and normative development.

Beyond these applications, a substantial body of work has examined the psychometric properties of the REI-40 across languages and cultures. The instrument has been validated, among others, in Sweden (Björklund & Bäckström, 2008), the Netherlands (Witteman et al., 2009), Argentina (Reyna & Ortiz, 2016), and Serbia (Purić & Jokić, 2023). Across these studies, internal-consistency estimates have generally been acceptable to good. The factorial evidence, however, has been more heterogeneous: although several studies support the four correlated-factor structure, others report higher-order or two-factor solutions (e.g., Reyna & Ortiz, 2016), and the incremental fit indices vary appreciably across samples. Notably, Björklund and Bäckström (2008) explicitly compared the hypothesized structure against alternative models—including single-factor and two-factor solutions—and found the multifactor structure superior, a model-comparison strategy adopted in the present study precisely because the dimensionality of the REI-40 remains an open question. This accumulated evidence underscores both the cross-cultural robustness of the REI-40 and the importance of population-specific validation, including the use of estimators and reliability indices appropriate to the ordinal nature of the four-point response scale.

### 1.3. The Brazilian Context and Professional Sample

Brazil’s unique cultural context provides an important test case for the REI-40’s cross-cultural validity. As South America’s largest country, with over 210 million inhabitants, Brazil represents a significant share of the Portuguese-speaking population worldwide. The validation of psychological instruments for Brazilian Portuguese is essential, given linguistic and cultural differences from European Portuguese that may affect item interpretation and response patterns.

The Advocacy-General of the Union (AGU) serves as Brazil’s federal legal representative, handling judicial and extrajudicial defense of the federal government while providing legal advisory services to the Executive Branch. AGU professionals are selected through highly competitive public examinations that emphasize analytical reasoning and legal knowledge. These professionals engage in complex decision-making that requires both systematic legal analysis and intuitive judgment about case strategies, client relations, and policy implications. This professional context provides an ideal population for examining thinking style preferences and their measurement properties in high-functioning individuals.

It is important to note that in Brazil, civil servants are selected through public competitive examinations that typically have more than 100 candidates per vacancy. Thus, the sample represents individuals who have demonstrated strong analytical capabilities through this selection process. This characteristic makes the sample particularly interesting for examining the measurement of rational thinking styles and for assessing whether experiential processing dimensions can be reliably measured in analytically oriented professionals.

### 1.4. Study Aims and Hypotheses

This study aimed to provide a comprehensive psychometric evaluation of the REI-40 for Brazilian Portuguese. Specifically, we sought to: (a) test the hypothesized four-factor structure through confirmatory factor analysis; (b) assess internal consistency reliability for each subscale; and (c) examine convergent and discriminant validity using the Cognitive Reflection Test (CRT-7). Based on dual-process theory and previous validation studies, we hypothesized that: (1) the four-factor structure would demonstrate acceptable fit to the data; (2) all subscales would show adequate internal consistency (ω ≥ 0.70); (3) rational subscales would correlate positively with CRT-7 performance (convergent validity); and (4) experiential subscales would show weak or negative correlations with CRT-7 (discriminant validity).

## 2. Method

### 2.1. Participants

The sample comprised 464 legal professionals from Brazil’s Advocacy-General of the Union (AGU). Demographic characteristics are presented in Table 1. The sample was predominantly male (52.4%), with a mean age of 47.1 years (SD = 10.0, range = 19–81). Most participants held bachelor’s degrees (69.0%), with 23.3% holding master’s degrees and 4.7% doctoral degrees. Regarding race/ethnicity, 69.4% self-identified as White, 22.2% as Pardo (mixed race), and 5.0% as Black.

This sample size exceeded recommended guidelines for confirmatory factor analysis, which suggest a minimum of 200 participants or a ratio of at least 10 participants per estimated parameter (Kline, 2016). With 40 items and four factors, our sample yielded an approximately 12:1 ratio, ensuring adequate statistical power for the planned analyses. The Kaiser-Meyer-Olkin measure of sampling adequacy was 0.89, and Bartlett’s test of sphericity was significant (*p* < 0.001), confirming the data’s suitability for factor analysis.

### 2.2. Measures

Rational-Experiential Inventory (REI-40). The 40-item REI-40 (Pacini & Epstein, 1999) was translated into Brazilian Portuguese using standard back-translation procedures. The original English version was translated into Brazilian Portuguese by the authors, who are fluent in both languages, then back-translated into English by an independent translator. Discrepancies were resolved through consensus between the authors and the back-translator. Participants rated items on a 4-point Likert scale (1 = Completely False to 4 = Completely True). The instrument yields four subscale scores: Rational Ability (10 items), Rational Engagement (10 items), Experiential Ability (10 items), and Experiential Engagement (10 items). The complete Brazilian Portuguese version of the REI-40, including all items and scoring instructions, is available from the corresponding author upon request, and the full item set can also be found in Appendix A.

Content validation was conducted by six expert judges in the behavioral sciences, who evaluated items for clarity, semantic equivalence, and cultural appropriateness on a 1–5 scale. The Content Validity Index (CVI) was calculated, with scores of 4 or higher considered adequate. Items with a CVI ≥ 0.80 were considered valid according to established guidelines (Alexandre & Coluci, 2011). All 40 translated items met this criterion, supporting the content validity of the Brazilian Portuguese version.

Cognitive Reflection Test (CRT-7). The seven-item version of the Cognitive Reflection Test (Toplak et al., 2014) served as a criterion for validity. The CRT-7 measures analytical thinking by presenting problems that evoke intuitive but incorrect responses, requiring deliberate reasoning to identify correct answers. For example, one item presents a scenario where the intuitive answer conflicts with the mathematically correct answer. The CRT-7 has demonstrated strong psychometric properties and is widely used to measure reflective versus intuitive cognitive style. Based on dual-process theory, we hypothesized that rational REI-40 subscales would correlate positively with CRT-7 performance, while experiential subscales would show negative or weak correlations.

### 2.3. Procedure

Data collection utilized Microsoft Forms to ensure participant anonymity and data security. The AGU Information Technology Department administered the survey via email invitation to all agency members, with responses automatically anonymized before being transferred to research personnel. The survey was available for four weeks, with reminder emails sent at two-week intervals. After data transfer was completed, the original response records were permanently deleted to protect participant confidentiality.

All participants provided informed consent through the electronic survey system, which described the study’s purpose, procedures, voluntary nature of participation, and data protection measures. The study protocol received approval from the Ethics Compliance Committee for Research Involving Human Subjects at Fundação Getulio Vargas (FGV CEPH; Addendum Decision No. P.147.2024), ensuring compliance with Brazilian research ethics standards and Resolution 466/2012 of the National Health Council.

### 2.4. Statistical Analysis

Confirmatory factor analysis (CFA; Brown, 2015) was conducted in R version 4.4.2 with the lavaan package (Version 0.6-20) (Rosseel, 2012). Because the REI-40 items use a four-category ordinal response scale, the confirmatory models were estimated with the robust weighted least squares estimator (WLSMV), which treats the indicators as ordinal and is preferable to normal-theory maximum likelihood when items have few response categories (Li, 2016). A confirmatory rather than exploratory approach was adopted because the REI-40 has a well-established, theory-derived four-factor structure (Pacini & Epstein, 1999); the aim was to test this a priori measurement model in the Brazilian sample rather than to derive a structure inductively. Model fit was evaluated using multiple indices following current recommendations (Hu & Bentler, 1999; Marsh et al., 2004; Kline, 2016): Comparative Fit Index (CFI) and Tucker-Lewis Index (TLI) values ≥ 0.90 indicate acceptable fit and ≥0.95 excellent fit; Root Mean Square Error of Approximation (RMSEA) values ≤ 0.08 indicate acceptable fit and ≤0.05 good fit; Standardized Root Mean Square Residual (SRMR) values ≤ 0.08 indicate acceptable fit. The robust (scaled) versions of these indices are reported throughout.

To evaluate the four-factor structure against competing accounts, five models were compared: a unidimensional model, a two-factor model (Rationality and Experientiality), the hypothesized four-factor model, a higher-order model with two second-order factors, and a four-factor model with the between-system factor correlations fixed to zero. Models were compared using the scaled chi-square difference test and changes in the approximate fit indices. Measurement invariance across sex, educational level, and age (median split) was tested following the procedure of Wu and Estabrook (2016) for ordinal indicators, implemented with the semTools package(Version 0.5-7), by comparing configural, threshold, and loading (metric) invariance; invariance was evaluated primarily by ΔCFI ≤ 0.010 (Chen, 2007). Because the lowest response category was rarely endorsed, and empty in one group for several items, the two lowest categories were merged for the invariance analyses, yielding three-category indicators.

Internal consistency was assessed using McDonald’s omega (ω), estimated from the CFA solution, with values ≥ 0.70 considered acceptable and ≥0.80 good; omega was preferred over coefficient alpha because it does not assume tau-equivalence (i.e., equal loadings across items) and therefore provides a more accurate reliability estimate when loadings are heterogeneous. Convergent and discriminant validity were evaluated through Pearson correlations between REI-40 subscale scores and CRT-7 total scores. Effect sizes were interpreted following Cohen’s (1988) guidelines: r = 0.10 (small), r = 0.30 (medium), r = 0.50 (large).

## 3. Results

### 3.1. Preliminary Analyses

Before the main analyses, data were screened for missing values, outliers, and distributional properties. No missing data were present on REI-40 items. Univariate outliers were examined using standardized scores; no values exceeded |3.29|. Multivariate normality was assessed using Mardia’s coefficient, which indicated some departure from multivariate normality; together with the ordinal nature of the four-point items, this supported the use of the WLSMV estimator for the confirmatory analyses.

### 3.2. Confirmatory Factor Analysis and Model Comparison

CFA of the hypothesized four-factor, 40-item model, estimated with WLSMV, demonstrated acceptable fit: χ^2^(734) = 1815.8, CFI = 0.902, TLI = 0.896, RMSEA = 0.056 (90% CI [0.053, 0.060]), SRMR = 0.080. All factor loadings were statistically significant (*p* < 0.001). Three items showed comparatively weak loadings, namely RA2 (λ = 0.122), RE8 (λ = 0.265), and EA4 (λ = 0.230), whereas all remaining loadings were substantial (range 0.42–0.85). Following the principle that a consolidated, internationally used instrument should be retained intact whenever possible (cf. Witteman et al., 2009), and because the full 40-item model already met conventional fit criteria under the ordinal estimator, all 40 items were retained; the three weaker items are considered further in the Discussion. Table 2 presents the fit indices for the 40-item model alongside the competing structural models.

Whereas an earlier specification estimated using normal-theory maximum likelihood yielded CFI and TLI values below the conventional 0.90 threshold, treating the items as ordinal with the WLSMV estimator raised the incremental fit indices to acceptable levels (CFI = 0.902, TLI = 0.896). This improvement is expected because maximum-likelihood estimation is less appropriate than categorical estimators for items with few response categories (Li, 2016). The RMSEA (0.056) and SRMR (0.080) likewise met established criteria, and the chi-square-to-degrees-of-freedom ratio (χ^2^/df = 2.47) fell below the conventional value of 3.0, together indicating acceptable absolute and incremental fit for the four-factor model.

Table 3 and Table 4 present the complete standardized factor loadings for all 40 items. All loadings were statistically significant (*p* < 0.001). Loadings for Rational Ability ranged from 0.122 to 0.828, with RA8 showing the highest loading and RA2 the lowest. Loadings for Rational Engagement ranged from 0.265 to 0.838, with RE6 showing the highest loading and RE8 the lowest. Loadings for Experiential Ability ranged from 0.230 to 0.852, with EA9 showing the highest loading and EA4 the lowest. Loadings for Experiential Engagement ranged from 0.513 to 0.853, with uniformly substantial values across items.

To situate the four-factor model among competing accounts of the REI-40’s structure, five models were estimated and compared (Table 2). A unidimensional model fit the data poorly (CFI = 0.468), as did a two-factor model distinguishing only Rationality from Experientiality (CFI = 0.861), indicating that neither a single general factor nor a coarse two-system structure adequately represents the data. The hypothesized four-factor model (CFI = 0.902) fit significantly better than both (scaled Δχ^2^ tests, *p* < 0.001), confirming that the ability and engagement facets within each system are empirically distinguishable. A higher-order model with two second-order factors fit slightly less well than the four-factor model (CFI = 0.892; Δχ^2^(3) = 47.2, *p* < 0.001), and a model fixing the between-system correlations to zero, although descriptively comparable (CFI = 0.922), was rejected by the scaled difference test (Δχ^2^(4) = 17.5, *p* = 0.002). Together, these comparisons support the four correlated factors as the most appropriate representation of the data, with strong within-system and weak between-system associations.

### 3.3. Factor Correlations

Factor correlations (Table 5) supported the dual-process theoretical structure. Strong positive correlations were found within the rational system (RA–RE: r = 0.688, *p* < 0.001) and within the experiential system (EA–EE: r = 0.787, *p* < 0.001). These high within-system correlations indicate that ability and engagement dimensions cohere within each processing mode, as predicted by CEST. Between-system correlations were weak (r = −0.10 to 0.20), supporting the relative independence of rational and experiential processing; the strongest between-system association (RA–EA = 0.195) remained modest, and the RE–EE correlation was non-significant. This pattern—substantial covariation within each system and only weak covariation across systems—is consistent with the dual-process conception of two largely autonomous processing modes.

### 3.4. Internal Consistency

Internal consistency, estimated with McDonald’s omega from the four-factor model, was good for all subscales: Rational Ability (ω = 0.80), Rational Engagement (ω = 0.85), Experiential Ability (ω = 0.79), and Experiential Engagement (ω = 0.87). All subscales met or exceeded the criterion for good reliability (ω ≥ 0.79). Omega was preferred over coefficient alpha because it does not assume tau-equivalence; this distinction was not merely formal, as alpha and omega diverged most for Rational Ability (α = 0.72 vs. ω = 0.80), where the heterogeneity of the loadings—including the weak RA2 item—led alpha to underestimate reliability. The omega estimates, therefore, provide a more accurate picture of the internal consistency of the Brazilian Portuguese REI-40.

### 3.5. Convergent and Discriminant Validity

Table 5 presents correlations between REI-40 subscales and CRT-7 scores. Convergent validity was supported by significant positive correlations between the rational subscales and CRT-7 performance: Rational Ability showed a medium effect size (r = 0.292, *p* < 0.001), and Rational Engagement showed a small-to-medium effect size (r = 0.255, *p* < 0.001). These findings indicate that individuals who report higher rational thinking ability and engagement also perform better on tasks requiring analytical reasoning, providing evidence that the rational subscales measure constructs related to analytical cognitive processing.

Discriminant validity was supported by weak, non-significant, or near-zero correlations between the experiential subscales and CRT-7: Experiential Ability showed a small, non-significant negative correlation (r = −0.077, *p* = 0.096), and Experiential Engagement showed a small but significant negative correlation (r = −0.119, *p* = 0.010). These patterns align precisely with theoretical predictions, demonstrating that experiential processing represents a distinct cognitive style rather than simply the inverse of rational processing. The weak negative correlations suggest that while experiential thinking is largely independent of analytical performance, individuals who strongly prefer intuitive processing may be slightly less likely to engage in the deliberate reasoning required by the CRT.

### 3.6. Measurement Invariance

Measurement invariance was examined across sex, educational level (postgraduate degree vs. no postgraduate degree), and age (median split at 46 years), following the procedure of Wu and Estabrook (2016) for ordinal indicators. Because the lowest response category was rarely used and empty in one group for several items, the two lowest categories were merged for these analyses, yielding three-category indicators. Table 6 reports the fit of the configural, threshold-invariant, and loading-invariant (metric) models.

For all three grouping variables, the configural model fit well (CFI = 0.909–0.0917), indicating that the four-factor configuration is supported within each subgroup. Imposing equal thresholds and then equal loadings produced negligible change in fit: the change in CFI between the configural and the metric models was ≤0.001 in every case, well within the ΔCFI ≤ 0.010 criterion for invariance (Chen, 2007). The scaled chi-square difference test was non-significant for education (*p* = 0.058) and age (*p* = 0.092); for sex, it was significant (*p* = 0.006), but, given the negligible ΔCFI, this most plausibly reflects the well-known oversensitivity of the chi-square test at this sample size rather than substantively meaningful non-invariance. Overall, the REI-40 demonstrated configural and metric invariance across sex, education, and age, supporting comparisons of thinking-style scores across these demographic groups. 

## 4. Discussion

This study provides the first comprehensive psychometric validation of the REI-40 for Brazilian Portuguese. The results support the four-factor structure predicted by Cognitive-Experiential Self-Theory, with evidence for adequate reliability and construct validity in a sample of legal professionals. These findings extend the cross-cultural validity of the REI-40 and provide Brazilian researchers and practitioners with a validated instrument for assessing individual differences in rational and experiential thinking styles.

### 4.1. Factor Structure and Model Fit

The confirmatory factor analysis supports the hypothesized four-factor structure. When the four-point items were treated as ordinal and the model was estimated with WLSMV, the incremental fit indices reached conventional criteria for acceptable fit (CFI = 0.902, TLI = 0.896), and the RMSEA (0.056) and SRMR (0.080) indicated acceptable absolute fit. This contrasts with an earlier normal-theory specification, in which the incremental indices fell below 0.90 because maximum-likelihood estimation is less suitable than categorical estimation for items with few response categories (Li, 2016). The model comparison reinforces this conclusion: the four-factor structure clearly outperformed unidimensional and two-factor alternatives, replicating, in a Brazilian sample, the pattern reported by Björklund and Bäckström (2008) in their Swedish validation.

All 40 items were retained. Three items (RA2, RE8, and EA4) showed comparatively weak standardized loadings, as also observed in the original instrument and in other validations (Pacini & Epstein, 1999; Witteman et al., 2009). Rather than removing them, we retained the full item set, both to preserve comparability with the original REI-40 and international studies and because the complete 40-item model already met conventional fit criteria under the ordinal estimator. Notably, two of these three items (RA2 and EA4) are reverse-scored; weak loadings for reverse-worded items are a well-documented manifestation of method (wording) variance rather than necessarily a substantive failure of the construct, and this interpretation is preferable to attributing the pattern to a deficiency of the items themselves. Future research may nonetheless examine whether minor rewording improves their performance in Brazilian samples.

The measurement invariance analyses further support the structural validity of the Brazilian Portuguese REI-40. Configural and metric invariance held across sex, educational level, and age, indicating that both the four-factor configuration and the item–factor relationships are equivalent across these groups. This addresses a key requirement for instruments used in heterogeneous samples and provides initial evidence—within a single national context—that the REI-40 measures thinking styles comparably across demographic subgroups, although invariance across languages and cultures remains to be established.

### 4.2. Support for Dual-Process Theory

The pattern of factor correlations provides strong support for CEST’s dual-process framework. The high within-system correlations (RA-RE: r = 0.679; EA-EE: r = 0.762) indicate that ability and engagement dimensions cohere within each processing mode, suggesting that confidence in one’s thinking abilities is related to one’s preference for using that type of thinking. The weak between-system correlations (r = −0.122 to 0.141) support the theoretical independence of rational and experiential systems, indicating that an individual’s rational processing style is largely unrelated to their experiential processing style. This pattern mirrors findings from other REI-40 validations internationally (Pacini & Epstein, 1999; Witteman et al., 2009) and provides evidence for the cross-cultural validity of the dual-process construct.

### 4.3. Validity Evidence

The validity evidence from CRT-7 correlations aligns precisely with theoretical predictions, supporting both convergent and discriminant validity. The positive correlations between rational subscales and CRT-7 performance (r = 0.255–0.292) indicate that individuals with higher rational preferences perform better on tasks requiring deliberate, effortful reasoning. These effect sizes are consistent with the theoretical expectation that self-reported thinking preferences should be related to, but not identical with, actual cognitive performance. The moderate magnitude of these correlations suggests that, while the REI-40 rational subscales capture meaningful aspects of analytical processing, they assess self-perceptions and preferences rather than cognitive ability per se.

The weak-to-negative correlations between experiential subscales and CRT-7 (r = −0.077 to −0.119) provide important discriminant validity evidence. These findings indicate that experiential processing represents a genuinely distinct cognitive style rather than merely the absence of rational processing. Individuals who rely heavily on intuition and feelings in their thinking are not simply lacking in analytical ability; rather, they possess and prefer a different mode of processing information. This pattern supports the CEST conceptualization of two parallel systems rather than a single rational-intuitive continuum.

### 4.4. Limitations and Future Directions

Several limitations should be acknowledged when interpreting these findings. First, the sample of legal professionals, while providing important evidence for workplace applications, may not generalize to the broader Brazilian population. Legal professionals are selected through competitive examinations emphasizing analytical skills and represent a highly educated, professional group. Future research should examine the REI-40’s performance across diverse educational, socioeconomic, and occupational groups within Brazil to establish broader population norms and examine measurement invariance across groups.

Second, although measurement invariance was established across sex, educational level, and age within the Brazilian sample, invariance relative to the original English version was not tested. Such cross-language analyses would require access to comparable samples from the original validation population and represent an important avenue for future research that would allow meaningful cross-cultural comparisons of thinking-style profiles.

Third, criterion-related validity was limited to the CRT-7, providing evidence of relationships with analytical thinking but not with behaviorally relevant outcomes. Future research should examine relationships between REI-40 scores and criteria such as job performance, decision quality, academic achievement, or health behaviors to establish the instrument’s predictive utility in applied contexts.

Fourth, the self-report nature of the REI-40 may be influenced by social desirability and self-presentation biases. The moderate correlations with CRT-7 performance suggest that self-reported preferences may not always align with actual cognitive behavior. Longitudinal studies and research incorporating behavioral measures of thinking style would strengthen the understanding of the construct’s validity.

Finally, three items (RA2, RE8, and EA4) showed comparatively weak loadings. Although the full 40-item model met conventional fit criteria and all items were therefore retained, future research might examine alternative model specifications—such as bifactor models accounting for both general and specific thinking-style factors—or refined item wording, particularly for the reverse-scored items, to further strengthen the instrument.

## 5. Conclusions

This research establishes the REI-40 as a reliable and valid instrument for measuring rational and experiential thinking styles in Brazilian populations. The validation demonstrates that the fundamental constructs of Cognitive-Experiential Self-Theory translate effectively to the Brazilian context, with factor correlations and validity evidence consistent with the theoretical framework. The strong internal consistency and meaningful relationships with analytical task performance support the instrument’s use in research examining individual differences in cognitive processing.

The Brazilian Portuguese REI-40 shows promise for applications in organizational, educational, and clinical settings. In organizational contexts, the instrument may inform personnel selection, team composition, and leadership development. In educational settings, understanding students’ thinking style preferences can inform pedagogical approaches and learning support strategies. In clinical practice, the REI-40 can enhance assessment of cognitive style and inform intervention approaches.

Building on the evidence of measurement invariance across sex, education, and age reported here, future research should extend validation to broader Brazilian populations, test invariance across languages and cultures, and investigate relationships with behaviorally relevant outcomes. Despite the noted limitations, this validation provides Brazilian researchers and practitioners with a psychometrically sound tool for assessing individual differences in rational and experiential thinking styles.

## Figures and Tables

**Table 1:** Sample Demographic Characteristics (N = 464).

Characteristic	Value
Age (M ± SD)	47.1 ± 10.0 years
Age range	19–81 years
Gender	
Male	52.4%
Female	47.6%
Education	
Bachelor’s degree	69.0%
Master’s degree	23.3%
Doctoral degree	4.7%
Race/Ethnicity	
White	69.4%
Pardo (Mixed)	22.2%
Black	5.0%

**Table 2:** Fit Indices for the Four-Factor Model and Competing Structural Models (WLSMV estimation).

Model	χ^2^	df	CFI	TLI	RMSEA	SRMR
M1. Unidimensional	6623.5	740	0.468	0.439	0.131	0.196
M2. Two-factor	2270.5	739	0.861	0.854	0.067	0.095
M3. Four-factor (hypothesized)	1815.8	734	0.902	0.896	0.056	0.080
M4. Higher-order (two 2nd-order factors)	1933.8	737	0.892	0.885	0.059	0.087
M5. Four-factor, no between-system correlations	1605.3	738	0.922	0.917	0.050	0.087

**Table 3:** Standardized Factor Loadings for Rational Subscales (RA and RE).

Rational Ability (RA)	Loading	Rational Engagement (RE)	Loading
RA1 (R)	0.614	RE1	0.641
RA2 †	0.122		
RA3 (R)	0.595	RE2 (R)	0.520
RA4	0.498	RE3	0.777
RA5	0.607	RE4 (R)	0.722
RA6 (R)	0.634	RE5	0.782
RA7	0.705	RE6 (R)	0.838
RA8 (R)	0.828	RE7 (R)	0.828
		RE8 †	0.265
RA9 (R)	0.647	RE9 (R)	0.565
RA10	0.574	RE10	0.420

Note. Standardized loadings from the four-factor model estimated with WLSMV; all loadings are significant at *p* < 0.001. (R) = reverse-scored item. † RA2, RE8, and EA4 showed comparatively weak loadings (<0.30); all 40 items were retained in the model (see Section 4).

**Table 4:** Standardized Factor Loadings for Experiential Subscales (EA and EE).

Experiential Ability (EA)	Loading	Experiential Engagement (EE)	Loading
EA1 (R)	0.598	EE1 (R)	0.513
EA2	0.691	EE2	0.853
EA3	0.681	EE3 (R)	0.547
EA4 (R) †	0.230	EE4 (R)	0.587
EA5	0.542	EE5	0.833
EA6 (R)	0.515	EE6 (R)	0.760
EA7	0.468	EE7 (R)	0.786
EA8 (R)	0.678	EE8	0.676
EA9	0.852	EE9	0.586
EA10	0.711	EE10	0.723

Note. Standardized loadings from the four-factor model estimated with WLSMV; all loadings are significant at *p* < 0.001. (R) = reverse-scored item. † RA2, RE8, and EA4 showed comparatively weak loadings (<0.30); all 40 items were retained in the model (see Section 4).

**Table 5:** Correlations Between REI-40 Subscales and CRT-7.

	RA	RE	EA	EE
RA	–			
RE	0.688 ***	–		
EA	0.195 ***	0.138 **	–	
EE	–0.103 *	–0.048	0.787 ***	–
CRT-7	0.292 ***	0.255 ***	–0.077	–0.119 *

Note. * *p* < 0.05; ** *p* < 0.01; *** *p* < 0.001. Values above involving RA–EE are standardized factor correlations from the four-factor WLSMV model; correlations with CRT-7 are Pearson correlations computed with observed subscale scores.

**Table 6:** Measurement Invariance of the REI-40 Across Sex, Education, and Age.

Grouping	Invariance Level	CFI	TLI	RMSEA	SRMR
Sex	Configural	0.909	0.903	0.050	0.098
	+Thresholds	0.909	0.903	0.050	0.098
	+Loadings (metric)	0.908	0.905	0.049	0.098
Education	Configural	0.917	0.911	0.048	0.097
	+Thresholds	0.917	0.911	0.048	0.097
	+Loadings (metric)	0.918	0.915	0.047	0.097
Age	Configural	0.911	0.905	0.050	0.098
	+Thresholds	0.911	0.905	0.050	0.098
	+Loadings (metric)	0.911	0.908	0.049	0.099

Note. All models were estimated with WLSMV on three-category indicators. Configural and threshold models coincide in degrees of freedom owing to the Wu and Estabrook (2016) identification for three-category items.

## Data Availability

The data used in this study are available from the corresponding author upon reasonable request.

## Appendix A. The 40 Items of the Brazilian Portuguese REI-40, by Subscale

The complete Brazilian Portuguese version of the Rational-Experiential Inventory is reproduced below. RA = Rational Ability; RE = Rational Engagement; EA = Experiential Ability; EE = Experiential Engagement. (R) denotes reverse-scored items. Items are answered on a four-point scale (1 = Completely false to 4 = Completely true).

**Item**	**Subscale**	**Brazilian Portuguese Wording**
RA1 (R)	RA	Não sou muito bom (boa) em resolver problemas complicados.
RA2	RA	Não tenho dificuldades para refletir cuidadosamente sobre as coisas.
RA3 (R)	RA	Não sou uma pessoa que fica analisando as coisas.
RA4	RA	Normalmente tenho razões claras e explicáveis para as minhas decisões.
RA5	RA	Usar a lógica geralmente funciona bem para mim ao solucionar problemas em minha vida.
RA6 (R)	RA	Refletir cuidadosamente sobre as coisas não é um dos meus pontos fortes.
RA7	RA	Eu tenho uma mente lógica.
RA8 (R)	RA	Não sou muito bom em resolver problemas que demandem uma análise lógica cuidadosa.
RA9 (R)	RA	Não raciocino bem sob pressão.
RA10	RA	Sou muito melhor em achar a solução lógica das coisas do que a maioria das pessoas.
RE1	RE	Eu prefiro problemas complexos a problemas simples.
RE2 (R)	RE	Saber a resposta sem entender o raciocínio que está por trás, para mim, é o suficiente.
RE3	RE	Gosto de resolver problemas que exijam bastante raciocínio.
RE4 (R)	RE	Não gosto muito de ficar pensando nas coisas.
RE5	RE	Eu gosto de desafios intelectuais.
RE6 (R)	RE	Tento evitar situações que demandem pensar profundamente nas coisas.
RE7 (R)	RE	Não gosto de ter que pensar muito.
RE8	RE	Gosto de pensar em termos abstratos.
RE9 (R)	RE	Pensar muito e por muito tempo sobre algo me dá pouca satisfação.
RE10	RE	Aprender novas maneiras de pensar seria muito atraente para mim.
EA1 (R)	EA	Se eu fosse confiar nos meus instintos, cometeria erros com frequência.
EA2	EA	Quando se trata de confiar nas pessoas, geralmente posso me basear em meus instintos.
EA3	EA	Eu creio que posso confiar nos meus palpites.
EA4 (R)	EA	Acho que meus palpites podem ser tanto precisos quanto imprecisos.
EA5	EA	Em geral eu consigo sentir se uma pessoa está certa ou errada mesmo sem saber explicar como eu sei disso.
EA6 (R)	EA	Minhas opiniões instintivas provavelmente não são tão boas quanto as da maioria das pessoas.
EA7	EA	Eu confio nas minhas primeiras impressões sobre as pessoas.
EA8 (R)	EA	Não sou muito intuitivo(a).
EA9	EA	Usar meus instintos geralmente funciona bem para mim ao solucionar problemas em minha vida.
EA10	EA	Quase nunca erro quando ouço minhas intuições mais profundas para encontrar uma resposta.
EE1 (R)	EE	Em geral eu não confio nos meus sentimentos para me ajudar a tomar decisões.
EE2	EE	Gosto de confiar nas minhas impressões intuitivas.
EE3 (R)	EE	Acho que é tolice tomar decisões importantes com base em sentimentos.
EE4 (R)	EE	Eu não gostaria de depender de ninguém que se descrevesse como intuitivo.
EE5	EE	Com frequência sigo os meus instintos ao decidir sobre alguma coisa.
EE6 (R)	EE	Não gosto de situações em que eu tenho que confiar na intuição.
EE7 (R)	EE	Não acho que seja uma boa ideia confiar na intuição para tomar decisões importantes.
EE8	EE	Acho que há momentos em que se deve confiar na intuição.
EE9	EE	Costumo usar meu coração como guia para minhas ações.
EE10	EE	A intuição pode ser uma forma muito útil de resolver problemas.
